# ACCELERATED BRAIN AGING IS ASSOCIATED WITH MORTALITY ACROSS RACE

**DOI:** 10.1093/geroni/igac059.2834

**Published:** 2022-12-20

**Authors:** Ramon Casanova, Andrea Anderson, Ryan Barnard, Keenan Walker, Timothy Hughes, Stephen Kritchevsky, Lynne Wagenknecht

**Affiliations:** Wake Forest School of Medicine, Winston-Salem, North Carolina, United States; Wake Forest School of Medicine, Winston-Salem, North Carolina, United States; Wake Forest School of Medicine, Winston-Salem, North Carolina, United States; National Institute of Health, Baltimore, Maryland, United States; Wake Forest School of Medicine, Winston-Salem, North Carolina, United States; Wake Forest School of Medicine, Winston-Salem, North Carolina, United States; Wake Forest School of Medicine, Winston-Salem, North Carolina, United States

## Abstract

There is an increasing interest in using machine learning and artificial intelligence to estimate chronological age using neuroimaging data. The gap between chronological age and estimated brain age (brain age gap, BAG) is used as a measure of accelerated/resilient brain aging. Accelerated brain aging has been associated with increased mortality risk. However, these reports are based on cohorts mostly composed by white individuals. Here we capitalized on the racially diverse nature of the Atherosclerosis Risk in Communities Study (ARIC) cohort to investigate associations of brain across race. We used brain MRI scans from 1172 cognitively normal ARIC participants that were collected at ARIC Visit 5. Of those 772 were White and 366 were African Americans. We used Cox regression models to investigate BAG values associations with mortality. There were 163 deaths (dw = 124 and daa = 39) over 8 years of follow-up. Participants were stratified by tertiles according to BAG values. We found that, compared to those individuals with BAG scores in the highest tertile (>=1.15), those who scored in the lowest tertile (<= -1.3 years) to be associated with significantly lower mortality among the White (HR=0.41, 95% CI, [0.26–0.66], p < 0.001) and Black (HR=0.43, 95% CI, [0.20–0.92], p = 0.03) participants after adjusting for age, race-center, sex, education, diabetes, smoking and hypertension. Our analyses show that our approach to estimate chronological age using high-dimensional elastic net regression, produces BAG values which are associated with mortality not only in White individuals but also in African Americans.

